# The increasing need for biomarkers in intensive care unit-acquired weakness - are microRNAs the solution?

**DOI:** 10.1186/s13054-015-0901-1

**Published:** 2015-04-22

**Authors:** Sebastian T Lugg, Phillip A Howells, David R Thickett

**Affiliations:** School of Clinical and Experimental Medicine, College of Medical and Dental Sciences, Centre for Translational Inflammation Research (CITR), University of Birmingham Laboratories, Queen Elizabeth Hospital Birmingham, Birmingham, B15 2TH UK

## Abstract

There is an increasing focus on intensive care unit-acquired weakness, its underlying mechanisms and therapeutic options. In this article we offer a commentary on the paper by Bloch and colleagues entitled 'MiR-181a: a potential biomarker of acute muscle wasting following cardiac surgery'. There is a need for biomarkers for intensive care unit-acquired weakness, not only in clinical practice but also in order to streamline future therapeutic trials. MicroRNAs are attractive biomarkers, and may have an important role in this disease. We highlight the significance of the authors’ finding of miR-181a, a novel plasma biomarker for the development of acute muscle wasting in post-operative cardiac surgery patients and discuss future research that is needed in this field following on from the study findings.

In this issue of *Critical Care*, Bloch and colleagues report on the utility of miR-181a estimation as a potential biomarker of acute muscle wasting following cardiac surgery [1]. Intensive care unit-acquired weakness (ICUAW) is an increasingly recognised consequence of critical illness. It is both a common and major contributor to prolonged ICU stay and impaired quality of life of the patients who survive to discharge from hospital [2]. The major risk factors for developing ICUAW include sepsis, systemic inflammatory response syndrome and multi-organ failure [3,4]. Other risk factors include uncontrolled hyperglycaemia [5] and the use of glucocortoids and neuromuscular blocking agents [3,6]. A key feature of the disease is the loss of muscle mass resulting from a shift in the dynamic balance of muscle protein synthesis and breakdown and a reduction in force generating capacity, which is associated with prolonged weaning and ICU stay and disability in survivors [7]. At present, the molecular mechanisms underlying the muscle atrophy of ICUAW are not fully understood. Recent work by Bloch and colleagues has suggested that along with growth factors, microRNAs may have an important role in ICUAW [8] and muscle phenotype and atrophy in chronic obstructive pulmonary disease [9,10].

A biomarker for ICUAW would be useful clinically not only for diagnosis, prognostication and to guide rehabilitation, but also in order to facilitate potential future drug or interventional trials. A microRNA is a small non-coding RNA molecule whose function is RNA silencing and post-transcriptional regulation of gene expression. MicroRNAs are potentially attractive as disease biomarkers. There is increasing published literature on the complex mechanisms of action of microRNAs, their stability in plasma and relating their presence to disease state and severity of disease progression [11]. Their role in malignancy as both potential biomarker and therapeutic target has been reviewed [12], and early therapeutic studies using microRNAs are underway in the field of rheumatology [13]. Bloch and colleagues hypothesised that certain plasma microRNAs could be biomarkers of acute muscle wasting. They measured plasma levels of selected microRNAs pre- and post-operatively from a prospective observational study of 42 patients undergoing high-risk cardiothoracic surgery. Of these patients, 55% have been previously described to develop muscle wasting defined by measuring a cross-sectional area of the rectus femoris by ultrasound.

Of the microRNAs studied, the authors found miR-181a was significantly higher on the second post-operative day in patients who developed sonographic muscle wasting at 1 week compared to those who did not. Physical strength and rehabilitation were not assessed. They calculated that a rise from baseline greater than 1.7 times in miR-181a had 91% specificity and 56% sensitivity for subsequent muscle wasting. The authors comment that the test's high positive predictive value (91%) could be used to identify those patients at risk, but could not exclude a diagnosis of ICUAW because of its low sensitivity.

At present, the biological role of miR-181a is not fully understood. It is expressed in muscle and elsewhere and has been shown to be essential for muscle regeneration and differentiation after tissue injury [14], and also has a role in the regulation of inflammatory pathways [15]. More research is needed into the role of miR-181a and indeed other microRNAs that may have roles in ICUAW. It is possible miR-181a does not, as suggested by Bloch and colleagues, represent damage itself, but may be promoting the regeneration of muscle following the surgical insult [1].

## Future directions

The paper by Bloch and colleagues shows preliminary evidence to suggest that mir-181a may be a potential biomarker for the development of acute muscle wasting in critically unwell patients. This is an intriguing possibility, not only to allow insight into the structural and functional changes of skeletal muscle during critical illness but also to allow better targeting of current and novel treatments, be that nutrition, physiotherapy, pharmacotherapy or external electrical stimulation. Further research is needed to validate miR-181a as a biomarker in the wider ‘unselected’ ICU populations and its role in the changes observed in skeletal muscle in critical illness. This paper provides an exciting glimpse into ICUAW and future directions for research.

## Abbreviation

ICUAW: Intensive care unit-acquired weakness
